# Mood states of active and insufficiently active adolescents related to sleep quality, gender, academic performance and guidelines for the post-COVID-19 scenario

**DOI:** 10.3389/fpsyg.2024.1494456

**Published:** 2024-11-01

**Authors:** Alexandro Andrade, Anderson D’Oliveira, Keyla Mara dos Santos, Lavinia Falese, Stefania Mancone, Pierluigi Diotaiuti, Luca Stabile, Giorgio Buonanno

**Affiliations:** ^1^Health and Sports Science Center – CEFID/Santa Catarina State University – UDESC, Florianópolis, Santa Catarina, Brazil; ^2^Laboratory of Sports and Exercise Psychology - LAPE, Florianópolis, Santa Catarina, Brazil; ^3^Department of Human Sciences, Society and Health / University of Cassino and Southern Lazio, Cassino, Frosinone, Italy; ^4^Department of Civil and Mechanical Engineering, University of Cassino and Southern Lazio, Cassino, Frosinone, Italy

**Keywords:** students, mood, exercise, mental health, physical activity

## Abstract

**Objective:**

To evaluate the impact of physical activity on the mood states of active and insufficiently active Italian adolescents and the relationships with sleep quality, sex, and academic performance, analyzing guidelines for the post-COVID-19 scenario.

**Methods:**

This is a cross-sectional, quantitative, and descriptive study. Data collection was carried out through an electronic questionnaire via Google Forms. Adolescent students from schools in the Lazio region, Italy, were evaluated.

**Results:**

In total, 437 Italian adolescents were included, with a mean age of 15.9 ± 1.37 years. The average time of exercise reported by the students was 277 min per week. Physical activity appears to be positively correlated with improved mood states, particularly in adolescents who engage in 150 min or more of physical activity per week, who exhibited notably higher levels of vigor. It was found that female adolescents presented a worse mood state, greater tension, depression, anger, fatigue, and mental confusion, and worse vigor when compared to boys and that adolescents who had poor sleep quality presented significantly worse mood state. Adolescents with an insufficient perception of academic performance demonstrated a significantly worse mood state, tension, depression, anger, and mental confusion, with a submerged mood profile when compared to adolescents who perceived themselves as excellent, with an iceberg profile. In the regression analysis, it was found that for each minute of physical activity practiced, the score in the state of vigor of the adolescents increased by 0.02 and mental confusion decreased by 0.002.

**Conclusion:**

From the present findings, we conclude that recommendations on PA practices for adolescents should be strengthened, as a way to mitigate possible damage to mood states, especially in the post-COVID-19 scenario. Future studies need to deepen the relationships between the mental health, sex, and academic performance of adolescents to strengthen positive mood states and elucidate information on the type, intensity, and dosage of physical activity to achieve mood benefits.

## Introduction

The COVID-19 pandemic has posed significant challenges to the mental and physical health of the global population, affecting diverse age groups and social groups (Xiong et al., 2020; Diotaiuti et al., 2021; D’Oliveira et al., 2022). One of these populations is adolescents and adults in school and academic stages, who are already going through a complex developmental transition (Costello et al., 2011), and have ended up being impacted by the additional challenges of the COVID-19 pandemic (Metherell et al., 2022). The city of Cassino, Italy, like many others around the world, was no different, has faced significant challenges during the period of the COVID-19 pandemic (Caffo et al., 2020; Diotaiuti et al., 2022). This unprecedented context has affected not only physical health but also emotional impacts, especially among adolescents and adults in school and academic stages in the region (Caffo et al., 2020).

Among the many difficulties faced by adolescents and adults at school and in the academic environment, the reduction in physical activity levels during the COVID-19 pandemic, due to social isolation periods, leaving them insufficiently active during these periods, negatively accentuated the mood states of adolescents, such as high levels of tension, anger, and mental confusion (Kang et al., 2021; Hossain et al., 2022). On the other hand, for an improved mental state, it would be recommended to maintain positive mood states, such as greater vigor, lower states of depressive mood, anger, and fatigue, for example (Kang et al., 2021).

Mood reflects a set of emotional, bodily, and behavioral states, of a momentary and variable nature, usually involving several intensities (Terry et al., 2003). Mood states directly impact the daily life of a population, through work, study, and physical and mental health (White et al., 2023). According to Samełko et al. (2018), mood can be positively affected by reducing stress, promoting well-being, and relieving and making routine challenges comfortable. On the other hand, a negative mood state is characterized by high levels of depression and tension, intensifying low levels of enthusiasm, efficiency, and productivity (Samełko et al., 2018), and can also impair academic performance (Spence et al., 2022).

In addition, mood is known to be associated with sleep quality. Studies show that negative mood states, such as confusion, fatigue, and tension impair sleep, and increased vigor reduces the likelihood of poor sleep (Andrade et al., 2019; Wang and Bíró, 2021). Importantly, considering the impact of sex steroids during puberty, adolescent girls may be more susceptible to sleep and mood swings (Brandt et al., 2021; Hazell et al., 2023).

Previous research has consistently shown that regular physical activity is associated with improved mood states in adolescents, as well as improved self-esteem and cognitive function (Wang et al., 2021; Santana et al., 2022; Jang et al., 2023; Dong, 2024), and the positive effects of exercise on adolescent physical health are widely known. Physical activity, a low-cost, non-pharmacological intervention, is characterized as a potentially beneficial alternative approach to improve mental state levels, as well as promote positive effects on mood states, being particularly attractive in the public health area (Haoran, 2021; Ye et al., 2022). Recent evidence has highlighted several biological and psychological mechanisms by which physical activity improves mood and mental well-being. On the biological front, physical activity stimulates the release of neurotransmitters such as endorphins and serotonin, which are essential for regulating mood and feelings of happiness. In addition, exercise modulates the hypothalamic–pituitary–adrenal axis, a central stress response system, helping regulate the body’s response to stress and reducing cortisol levels, often elevated in depressed mood states (Hwang et al., 2023; Martín-Rodríguez et al., 2024).

However, adolescent students are known to be a population at risk for high levels of sedentary behavior due to routine studies, excessive use of screens, and social context (Castro et al., 2020). A recent study seeking to understand the sedentary behaviors of students and their impact on mood states failed to observe a significant association between the variables in a relatively large sample isolated due to the COVID-19 pandemic (Kang et al., 2021). On the other hand, the literature has argued that students and adolescents spending more time on screens, or engaging in other sedentary behaviors, can lead these populations to worse levels of mental states (Asare and Danquah, 2015; Maras et al., 2015; Schuch et al., 2020; Stanton et al., 2020).

Given the growing concern over the mental health of adolescents, particularly in the wake of the COVID-19 pandemic, it is crucial to explore how lifestyle factors, such as physical activity and sleep quality, interact to influence mood states. While previous studies have explored the relationship between physical activity and mood states, limited research has focused on how sleep quality moderates this relationship in adolescents and adults in school and academic stages, particularly in the context of varying levels of physical activity.

It is important to highlight that the city of Cassino, Italy, was uncertain about whether to maintain social isolation or continue with its activities normally between May and July 2020, as were many other cities in different countries. Despite this, student and academic activities continued continuously during this period.

In this context, we investigated how the challenges imposed by COVID-19 affected the physical activity levels of these young people, and how such changes are related to emotional states. Understanding these dynamics is critical not only to deepen our knowledge of adolescent mood states during the pandemic, but also to develop strategies and interventions that promote adolescent mental and physical health in a post-COVID-19 setting. The current study aimed to evaluate the impact of physical activity on the mood states of active and insufficiently active Italian adolescents in the city of Cassino, Italy, during the pandemic and the relationships with sleep quality, sex, and academic performance, analyzing guidelines for the post-COVID-19 scenario. We hypothesized that adolescents with higher levels of physical activity would exhibit better mood states and that this relationship would be further strengthened by higher sleep quality.

## Methods

### Study design

This descriptive cross-sectional study with a quantitative approach was carried out using self-administered online questionnaires during the COVID-19 pandemic, between May and July 2020, a period characterized by a greater and lesser degree of social isolation, according to the restrictive measures of the Italian health authorities, respectively. The study was approved by the Institutional Review Board of the University of Cassino under code IRB_SUSS_09: 21/02/19. The data collected were: health-related; physical activity level; and mood states of the adolescents participating in the study. To ensure the validity of our findings, we employed a cross-sectional design with a sufficiently large sample size and controlled for confounding variables such as age, gender; and academic performance.

### Participants

The study population was composed of adolescent students from the Lazio region of Italy. Convenience sampling was carried out with students from Italian schools who agreed to participate in the study. The adolescents were not in social isolation at the time of data collection, but they were involved in the local context of a global pandemic. The inclusion criteria were students of both sexes, aged between 12 and 20 years, and regular students from public and private schools in several Italian cities in the state of Lazio, Italy. The sample was balanced in terms of sex and represented a range of backgrounds, ensuring a diverse and representative participant pool. Regarding the exclusion criteria for participants, those who did not complete the data collection form completely did not participate in this study.

### Sample size

The Gpower 3.1 software was used to estimate the sample size. The calculation was performed *a priori*. For this purpose, the Wilcoxon-Mann–Whitney test field (two groups) was selected, from the t-test family, using an effect size of 0.5, alpha of 0.05 and power of 0.95 (Luiz and Magnanini, 2000; Matias et al., 2019), resulting in a total sample of 184 individuals. Due to the scarcity of cross-sectional studies that assess the mood variable in adolescents, we chose to estimate the effect size using a combined approach, using previous studies, pilot studies, and theoretical estimates (Maciejewski et al., 2019; Lee, 2021).

### Procedures

Initially, prior contact was made with the schools to disseminate the research and the objectives and procedures of the research were explained. Students accessed the electronic Google Forms via links distributed through messaging apps or email. The participants were directed to the instructions for filling out and subsequently signing the Informed Consent Form (ICF) presented electronically. The ICF contained information on the purpose of the study, and explained to the students that participation was voluntary, and that they could withdraw from the study at any time. The study was conducted following ethical guidelines, with informed consent obtained from all participants and their guardians, and confidentiality maintained throughout. The confidentiality of the participants’ data was preserved in a database where it was not possible to observe the names of the participants since at the time of data collection the participants were identified by a number and the database was stored in a secure folder together with the researcher responsible for the research.

After completing and signing the ICF, the data collection began through an identification form, prepared by the researchers, to collect data on age, sex, city of residence, school year, weight, and height, as well as data regarding their perception of their academic performance, quality of health, and physical activity practice (in the context of school physical education and outside it). In a second moment of data collection, the participants answered the Brunel Mood Scale (BRUMS) (Italian version) adapted to the digital environment, which aimed to investigate the mood state of the participants. As a secondary variable, the quality of the adolescents’ sleep was evaluated, in order to observe its relationship with the mood state.

#### The Brunel mood scale (BRUMS) (Italian version)

The Brunel Mood Scale (BRUMS) (Italian version), was used to assess mood states (tension, depression, anger, vigor, fatigue, and confusion) (Rohlfs et al., 2008). The BRUMS consists of 24 questions, with answer options ranging from 0 (none) to 4 (extremely), depending on the mood at the time of the assessment. By adding up the answers to the questions relating to each construct, a score ranging from 0 to 16 is obtained for each mood state.

According to Brandt et al. (2016), the items of each subscale, according to BRUMS are: (a) Anger: irritated, angry, mad, grumpy (items 7, 11, 19, 22); (b) Confusion: confused, insecure, disoriented, indecisive (items 3, 9, 17, 24); (c) Depression: depressed, discouraged, sad, unhappy (items 5, 6, 12, 16); (d) Fatigue: exhausted, whacked, sleepy, tired (items 4, 8, 10, 21); (e) Tension: terrified, anxious, worried, tense (items 1, 13, 14, 18); (f) Vigor: excited, willing, energetic, alert (items 2, 15, 20, 23). In its validation, the scale showed good internal consistency, with Cronbach’s alpha values above 0.70 for all constructs. Positive mood is characterized by a high level of vigor (positive factor) and low levels of fatigue, tension, depression, confusion and anger (negative factors), and is considered an ideal model of positive mental health (Rohlfs et al., 2008). The BRUMS has been proven to be a valid and reliable tool for assessing mood states in adolescents (Andrade et al., 2019; Pereira et al., 2020; Brandt et al., 2021).

#### Pittsburgh sleep quality index (PSQI) (Italian version)

Developed by Buysse et al. (1989) and validated by Konrad and Lopes (2005) in abbreviated form, this is a self-administered questionnaire that assesses sleep quality and pattern through specific questions about the previous month. The results are grouped into seven components, such as subjective quality, latency, duration, usual efficiency, disorders, medication use, and daytime dysfunctions. The results are evaluated on a scale of 0–3, with 3 being the negative end of the scale. The values of the domains are summed to obtain an overall score ranging from 0 to 21, with higher scores indicating poorer sleep quality. The scale is categorized into good sleep quality (score 0–5 points) or poor sleep quality (score > 5). The PSQI presents high internal consistency (Cronbach’s *α* reliability coefficient = 0.83) and high test–retest reliability, with a product–moment correlation of Pearson’s global score between T1 and T2 of 0.85 (*p* < 0.001) (Buysse et al., 1989).

#### Physical activity

The time in minutes of physical activity of the adolescents was collected through the question: How many hours per week do you dedicate to exercise and sport? Participants were classified as inactive (exercised for less than 150 min per week) or active (exercised for at least 150 min or more per week). Physical activity levels were classified according to the recommendations of the World Health Organization (Bull et al., 2020).

### Data analysis and processing

Data were collected using Google Forms and later tabulated in *Microsoft Excel software.* Data analysis was performed using the *Statistical Package for the Social Sciences* (SPSS), version 20.0. Descriptive and inferential analyses were performed. Demographic characteristics, such as age, sex, nationality, weight, height, and weekly time of physical activity were described using means, percentages, and standard deviations. Participants were categorized based on their level of physical activity according to the recommendations of the World Health Organization (Bull et al., 2020), as physically active or inactive.

The analysis of the data distribution was performed using the Kolmogorov–Smirnov test. The *Mann–Whitney U* test was used to compare the moods of active and insufficiently active adolescents. Simple linear regression analysis was used to identify the influence of physical activity on mood variables. Linear regression was chosen mainly because the dependent variable is numerical. In simple linear regression, the model is adjusted to predict the outcome based on a single predictor variable. In this case, the outcome variable used was vigor and the predictor variable was physical activity. The independence of residuals was tested by Durbin-Watson. The significance level was set at *α* ≤ 0.05. Missing data were addressed using multiple imputation techniques to ensure the robustness of the statistical analyses.

## Results

The sample consisted of 437 students, with a mean age of 15.97 ± 1.37 years, predominantly female (50.8%), and Italian (98.2%). The mean weight of the participants was 62.7 (±13.2) kg and the mean height was 170 (±8.37) cm.

Table 1 presents the characterization of the sample in terms of Body Mass Index (BMI), perception of health quality, frequency of smoking and getting sick, use of medications, perception of sleep quality, self-reported academic performance, and time of physical activity.

**Table 1:** Characteristics related to health, physical activity characteristics and division into active and insufficiently active groups of adolescents.

	Adolescents (*n* = 437)	Active adolescents (*n* = 297)	Insufficiently active adolescents (*n* = 140)
Age (Mean ± SD)	15.97 ± 1.37	15.95 ± 1.37	16.03 ± 1.3
Weight (Mean ± SD)	62.7 ± 13.2	63.01 ± 13.05	62.26 ± 13.62
Height (Mean ± SD)	170.36 ± 8.37	171 ± 8.53	169.06 ± 7.87
BMI	*N (%)*	*N (%)*	*N (%)*
Low weight	60 (13.7%)	45 (15.2%)	15 (10.7%)
Normal Weight	320 (73.2%)	215 (72.4%)	105 (75%)
Overweight	38 (8.7%)	26 (8.8%)	12 (8.6%)
Obesity grade I	6 (1.4%)	5 (1.7%)	1 (0.7%)
Obesity grade II	1 (0.2%)	0	1 (0.7%)
Extreme Obesity	12 (2.7%)	6 (2.0%)	6 (4.3%)
Sex	*N (%)*	*N (%)*	*N (%)*
Female	222 (50.8%)	147 (49.5%)	75 (53.6%)
Male	215 (49.2%)	150 (50.5%)	65 (46.4%)
Perception of health quality	*N (%)*	*N (%)*	*N (%)*
Good very good	*319 (73%)*	154 (51.8%)	62 (44.3%)
Normal	*114 (26*.*1%)*	92 (31%)	39 (27.9%)
Weak/Bad	*4 (0*.*9%)*	51 (17.2%)	39 (27.8%)
Frequency of getting Sick	*N (%)*	*N (%)*	*N (%)*
Rarely/Never	300 (68.7%)	208 (70%)	92 (65.8%)
Sometimes	117 (26.8%)	76 (25.6%)	41 (29.3%)
Often	20 (4.6%)	13 (4.4%)	7 (5%)
How often you smoke	*N (%)*	*N (%)*	*N (%)*
Rarely/Never	386 (88.3%)	263 (88.5%)	123 (87.9%)
Sometimes	22 (5.0%)	12 (4%)	10 (7.1%)
Often	16 (3.7%)	15 (5.1%)	1 (0.7%)
Ever	13 (3.0%)	7 (2.4%)	6 (4.3%)
Continuous medication use	*N (%)*	N (%)	N (%)
Yes	44 (10.1%)	30 (10.1%)	14 (10%)
In	393 (89.9%)	267 (89.9%)	126 (90%)
Perception of sleep quality	*N (%)*	N (%)	N (%)
Good/Excellent	157 (36%)	118 (39.7%)	39 (27.9%)
Normal	150 (34.3%)	100 (33.7%)	50 (35.7%)
Bad/Very bad	130 (29.7%)	79 (26.6%)	51 (36.4%)
Perception of academic performance			
Good/Excellent	203 (46.5%)	139 (46.8%)	64 (45.7%)
Normal	193 (44.2%)	132 (44.4%)	61 (43.6%)
Mediocre/Insufficient	41 (9.4%)	26 (8.7%)	15 (10.7%)
Minutes per week of exercise (Mean ± SD)	276.6 ± 217.6	370.65 ± 204.23	79 ± 43.91
Do you attend Physical Education classes?	*N (%)*	*N (%)*	*N (%)*
Yes	428 (97.9%)	294 (99%)	134 (95.7%)
No	9 (2.1%)	3 (1%)	6 (4.3%)

SD, standard deviation; F, absolute frequency; %, relative frequency/percentage.

Regarding the practice of exercises, 97.9% of the students reported participating in physical education classes. The weekly average number of minutes of physical activity both in and outside of physical education classes was 276.6 (±217.6) minutes. The study participants were categorized as physically active or inactive, according to the recommendations for physical activity levels of the World Health Organization (WHO). The majority (67.9%) of the sample reported physical activity levels greater than 150 min per week and were classified as active, while more than 32% of the adolescents did not comply with the WHO recommendations, with a weekly average of only 79 min of physical activity.

Table 2, in the comparison of the adolescents’ average vigor, shows that the active students (5.75 ± 3.49) had higher vigor when compared to the insufficiently active students (4.461 ± 3.20), with a significant difference between the average vigor values (*p* < 0.01). Considering the averages of the mood states tension, depression, anger, fatigue, and mental confusion, we can see a trend towards a better mood state among active adolescents, when compared to the averages of insufficiently active adolescents (Table 2).

**Table 2:** Mean values of the mood states of the 437 adolescents when comparing active and insufficiently active.

Mood	General mood (437)	Active adolescents (*n* = 297) Average ± DP	Insufficiently active adolescents (*n* = 140) Average ± DP	*p*-value
Tension	4.64 ± 3.97	4.48 ± 3.83	4.97 ± 4.25	0.38
Depression	2.64 ± 3.36	2.49 ± 3.35	2.96 ± 3.38	0.06
Anger	3.94 ± 3.75	3.93 ± 3.66	3.97 ± 3.94	0.76
Vigor	5.38 ± 3.43	5.75 ± 3.49	4.61 ± 3.20	0.00*
Fatigue	4.19 ± 3.86	4.04 ± 3.89	4.50 ± 3.80	0.12
Mental confusion	3.37 ± 3.71	3.36 ± 3.38	3.38 ± 3.44	0.37

SD, standard deviation; *statistical significance – Mann–Whitney *U* test.

Linear regression analysis showed that physical activity explains the increase in vigor in this population (*F* = 4.835, *p* = 0.03; *R*^2^ = 0.011), and for each minute of physical activity practiced, the score of the vigor domain increases by 0.02. Mental confusion also showed a significant relationship with physical activity in linear regression, indicating that for each minute of physical activity practiced, the score of this subscale decreases by 0.002 (*F* = 5.270; *p* = 0.022; *R*^2^ = 0.012).

The results of the regression were not significant for the anger subscales (*F* = 1.569; *p* = 0.211; *R*^2^ = 0.004), depression (*F* = 3.080; *p* = 0.080; *R*^2^ = 0.007), fatigue (*F* = 3.483; *p* = 0.063; *R*^2^ = 0.008) and tension (*F* = 3.774; *p* = 0.053; *R*^2^ = 0.009).

When comparing the mood between insufficiently active and active adolescents beyond the WHO recommendation (more than 300 min of moderate physical activity per week), it is possible to observe significant improvements in vigor (*p* < 0.01) and mental confusion (*p* = 0.05) (Table 3).

**Table 3:** Mean values of the mood states of adolescents when compared between insufficiently active and active beyond what is recommended by the WHO.

Mood	General mood (290)	Adolescents who are more active than recommended (*n* = 150) Average ± DP	Insufficiently active adolescents (*n* = 140) Average ± DP	*p*-value
Tension	4.57 ± 4.06	4.20 ± 3.85	4.97 ± 4.25	0.15
Depression	2.65 ± 3.29	2.36 ± 3.20	2.96 ± 3.38	0.07
Anger	3.81 ± 3.78	3.65 ± 3.63	3.97 ± 3.94	0.61
Vigor	5.35 ± 3.37	6.04 ± 3.39	4.61 ± 3.20	0.00*
Fatigue	4.25 ± 3.83	4.02 ± 3.86	4.50 ± 3.80	0.18
Mental confusion	3.07 ± 3.39	2.79 ± 3.33	3.38 ± 3.44	0.05*

SD, standard deviation; *statistical significance – Mann–Whitney *U* test.

In the comparison of mood state by sex, females presented a worse mood state than males (*p* < 0.01) (Table 4).

**Table 4:** Comparison of the mood of adolescents by sex.

Mood	Female (*n* = 222) Average ± DP	Male (*n* = 215) Average ± DP	P-value
Tension	5.99 ± 3.95	3.25 ± 3.49	0.00*
Depression	3.38 ± 3.63	1.88 ± 2.88	0.00*
Anger	4.85 ± 3.81	3.01 ± 3.45	0.00*
Vigor	4.72 ± 3.13	6.06 ± 3.60	0.00*
Fatigue	5.26 ± 3.95	3.08 ± 3.45	0.00*
Mental confusion	4.31 ± 3.96	2.40 ± 3.16	0.00*

SD, standard deviation; *statistical significance – Mann–Whitney *U* test.

**Figure 1:**
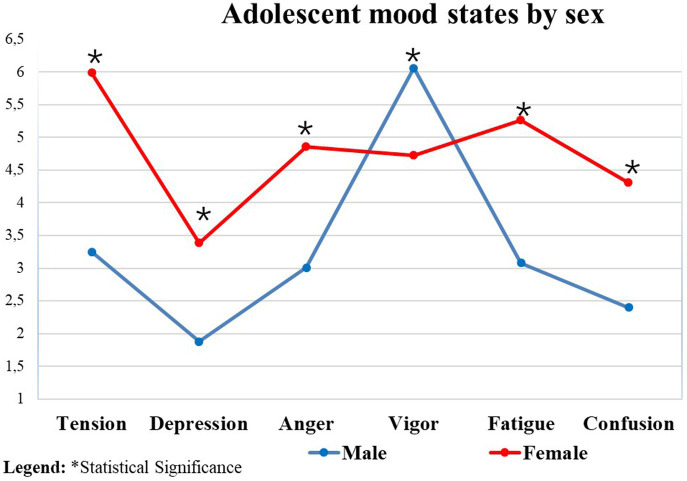
Mood status of adolescents compared by sex. *Statistical Significance.

Figure 1 shows the graph with the mean mood of the adolescents when compared by sex. We found that the mood of girls was significantly worse when compared to the mood of boys, presenting greater tension, depression, anger, fatigue, and mental confusion, and worse vigor when compared to boys.

When comparing the mood between good and poor sleep quality of adolescents, adolescents who reported a worse mood state presented significantly poorer sleep quality (*p* < 0.01) (Table 5).

**Table 5:** Comparison of the mood state of adolescents between the *Pittsburgh Sleep Quality Index (PSQI),* categorized by good and poor sleep quality.

Mood	Good sleep quality (*n* = 156) Average ± DP	Poor sleep quality (*n* = 281) Average ± DP	*p*-value
Tension	3.01 ± 3.12	5.54 ± 4.11	0.00*
Depression	1.18 ± 1.98	3.45 ± 3.69	0.00*
Anger	2.31 ± 2.70	4.85 ± 3.94	0.00*
Vigor	6.51 ± 3.77	4.75 ± 3.06	0.00*
Fatigue	2.54 ± 3.09	5.10 ± 3.95	0.00*
Mental confusion	1.82 ± 2.34	4.23 ± 4.04	0.00*

SD = standard deviation; *statistical significance – Mann–Whitney *U* test.

**Figure 2:**
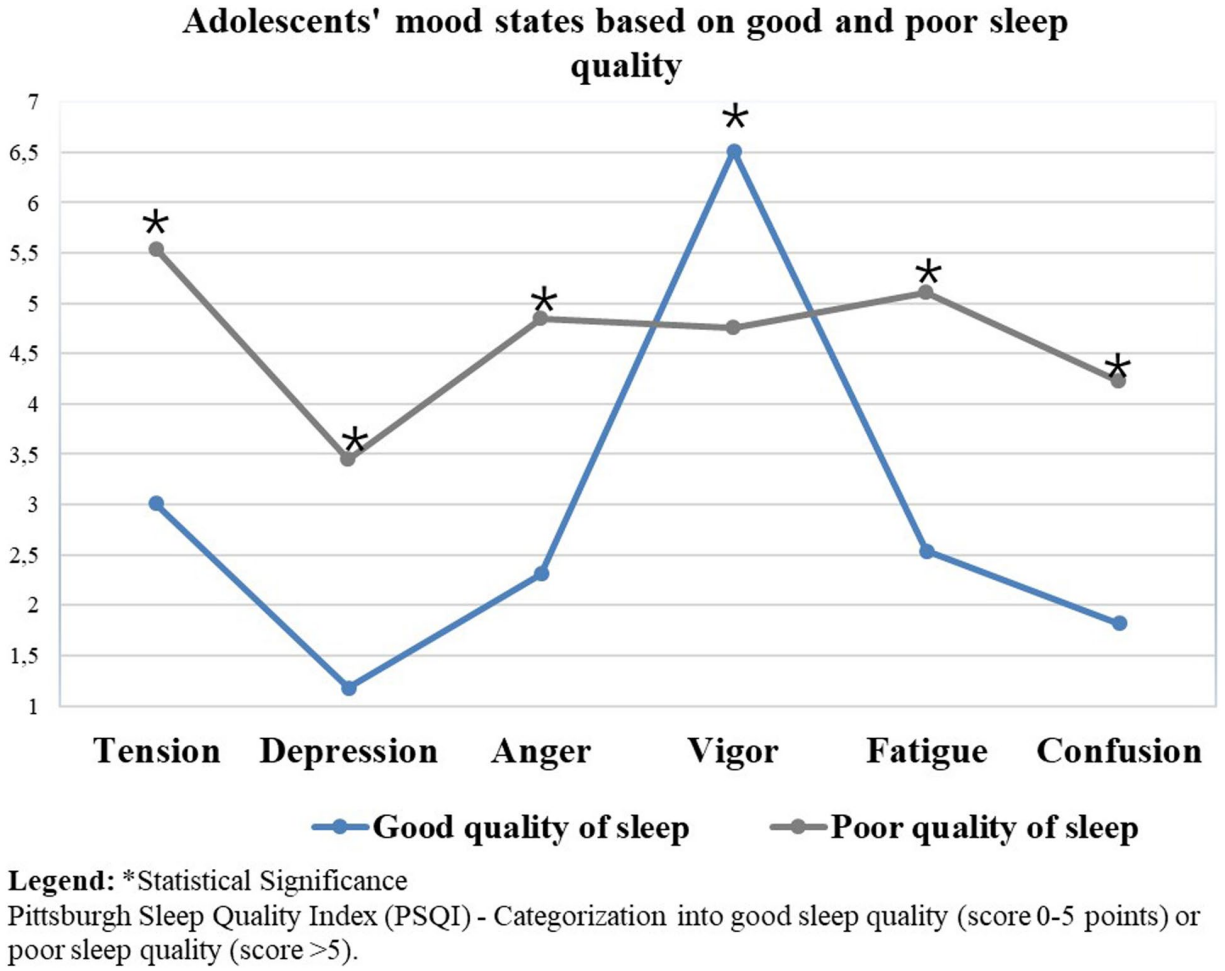
Mood status compared with good and poor sleep quality in adolescents. *Statistical Significance.

Figure 2 presents the iceberg graph with the mean mood of adolescents when comparing good and poor sleep quality among adolescents. We found that the differences in the mood states of adolescents with poor sleep quality were proportionally visible when compared to adolescents with good sleep quality.

**Figure 3:**
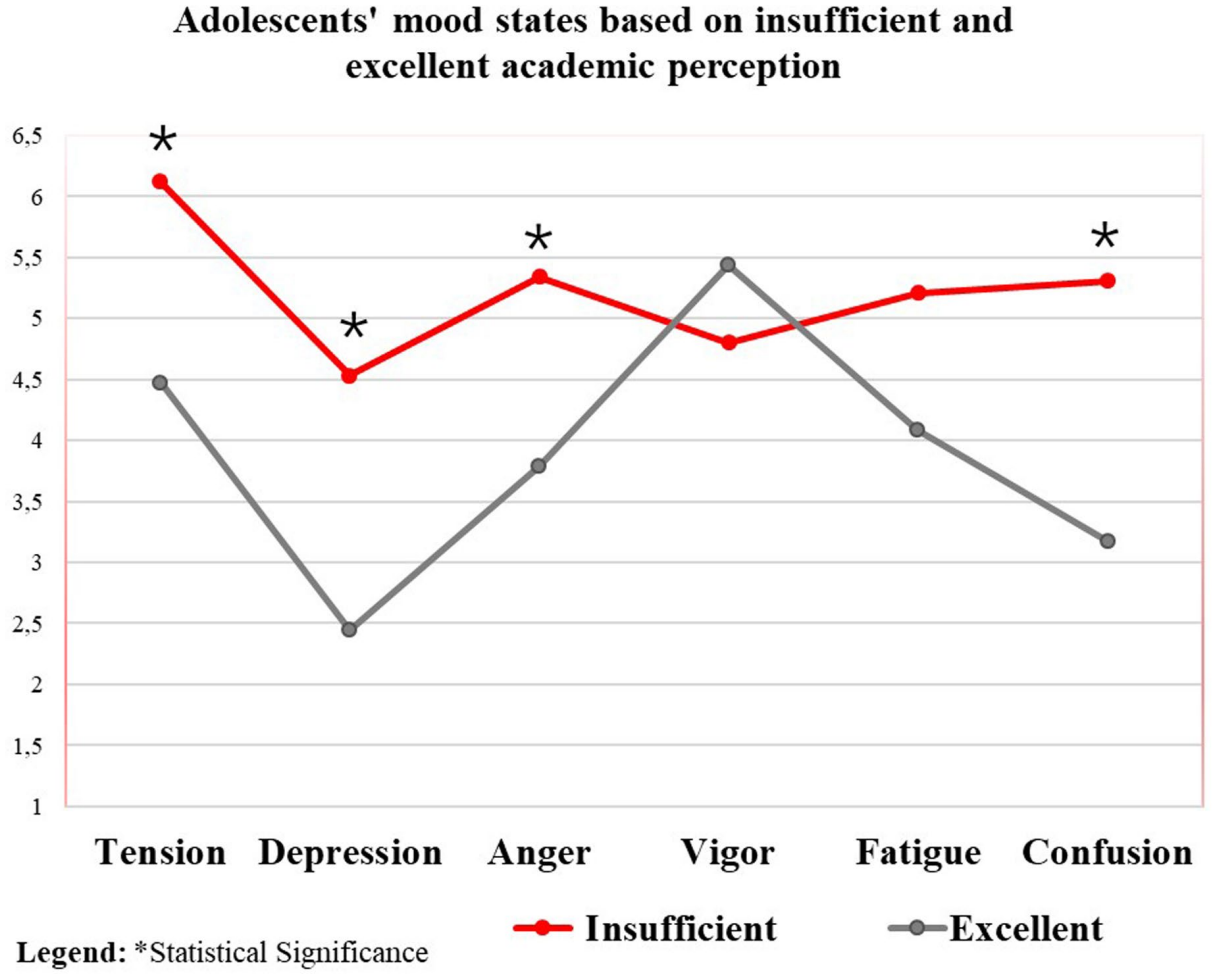
State of mood compared to insufficient and excellent academic perception. *Statistical Significance.

In the comparison between the mood states and the perception of academic performance of adolescents, we found that adolescents who perceive themselves as having insufficient academic performance report a significantly worse mood state with respect to tension, depression, anger, and mental confusion, when compared to adolescents with an excellent perception of their academic performance, characterized by the iceberg mood profile (Table 6). This negative profile can be characterized as a new mood profile, classified as submerged (Parsons-Smith et al., 2017). Figure 3 presents the graph based on the means of mood when comparing the perception of insufficient and excellent academic performance of adolescents.

**Table 6:** Comparison between the mood and the perception of academic performance (insufficient and excellent) of adolescents.

Mood	Insufficient (*n* = 41) Average ± DP	Excellent (*n* = 396) Average ± DP	*p*-value
Tension	6.12 ± 4.14	4.48 ± 3.93	0.00*
Depression	4.53 ± 4.36	2.44 ± 3.19	0.00*
Anger	5.34 ± 4.46	3.79 ± 3.64	0.01*
Vigor	4.80 ± 2.95	5.44 ± 3.48	0.28
Fatigue	5.21 ± 4.06	4.08 ± 3.83	0.06
Mental confusion	5.31 ± 4.82	3.17 ± 3.53	0.00*

SD, standard deviation; *statistical significance – Mann–Whitney *U* test.

**Figure 4:**
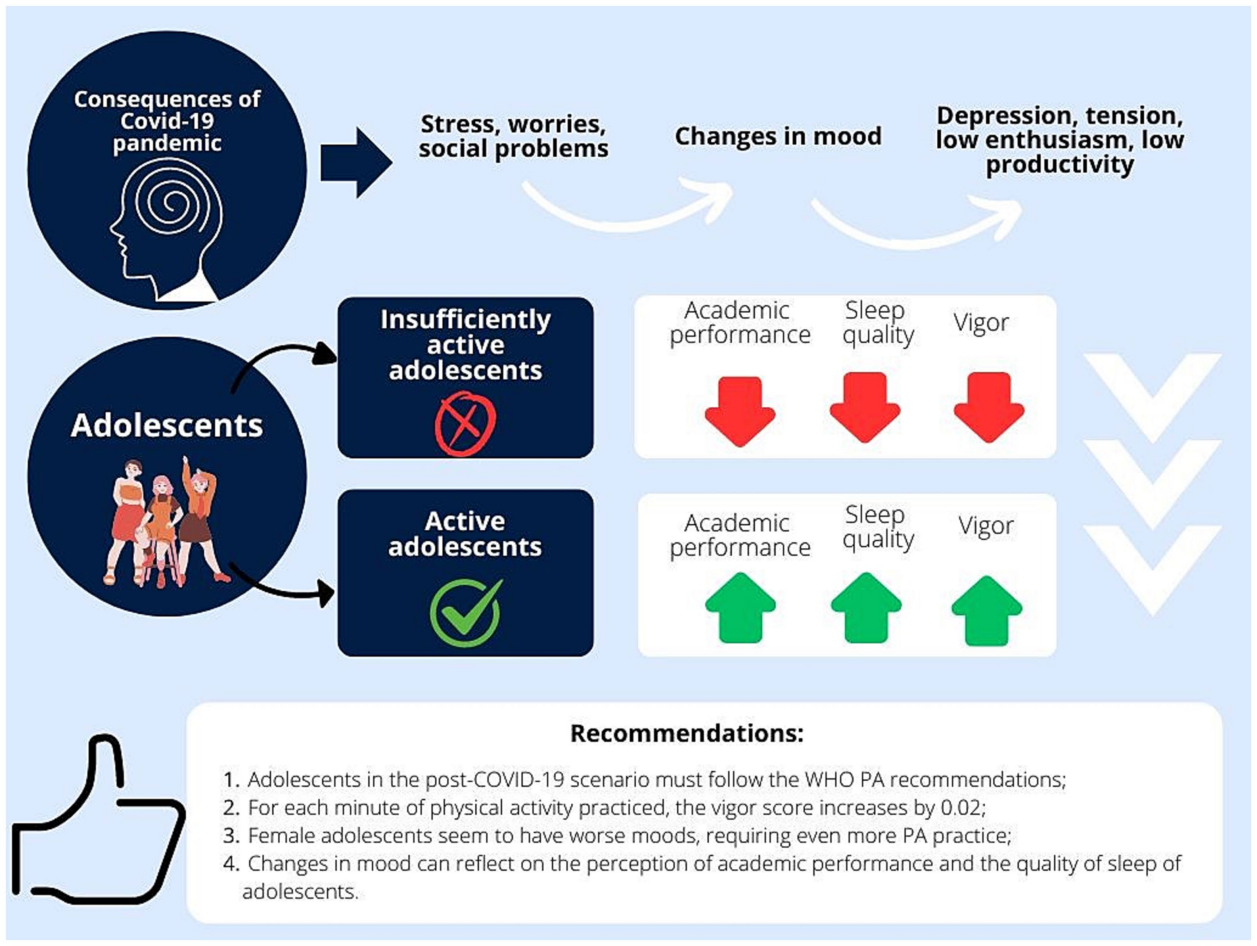
Recommendations for adolescents in the Post-COVID period.

Considering recommendations for physical activity in adolescents in the Post-Covid-19 scenario, we can highlight that adolescents should follow the WHO PA recommendations, practicing 150–300 min of moderate physical activity per week. With each minute of physical activity practiced, the adolescent’s state of vigor increases, showing significant improvements in mood, sleep, and academic performance. It is known that a pandemic scenario leads to increased levels of stress, worry, and social problems, reflecting in negative moods. Thus, physical activities act as a protective factor in adolescents in the post-COVID period (Figure 4).

As guidelines for adolescent health for the Post-COVID-19 period, there is a need to examine the mood profiles of adolescents and the effects of exercise interventions on the mental health of these individuals. Mood swings can lead to depressive disorders, low productivity, and low arousal in adolescents.

Regarding social confinement, the impact of the rigidity of social distancing measures on health conditions in other future pandemic situations needs to be better understood, investigated, and measured. Public health programs and future studies should especially address the needs of adolescents, as this is a population that is very likely to sustain these post-pandemic changes.

**Figure 5:**
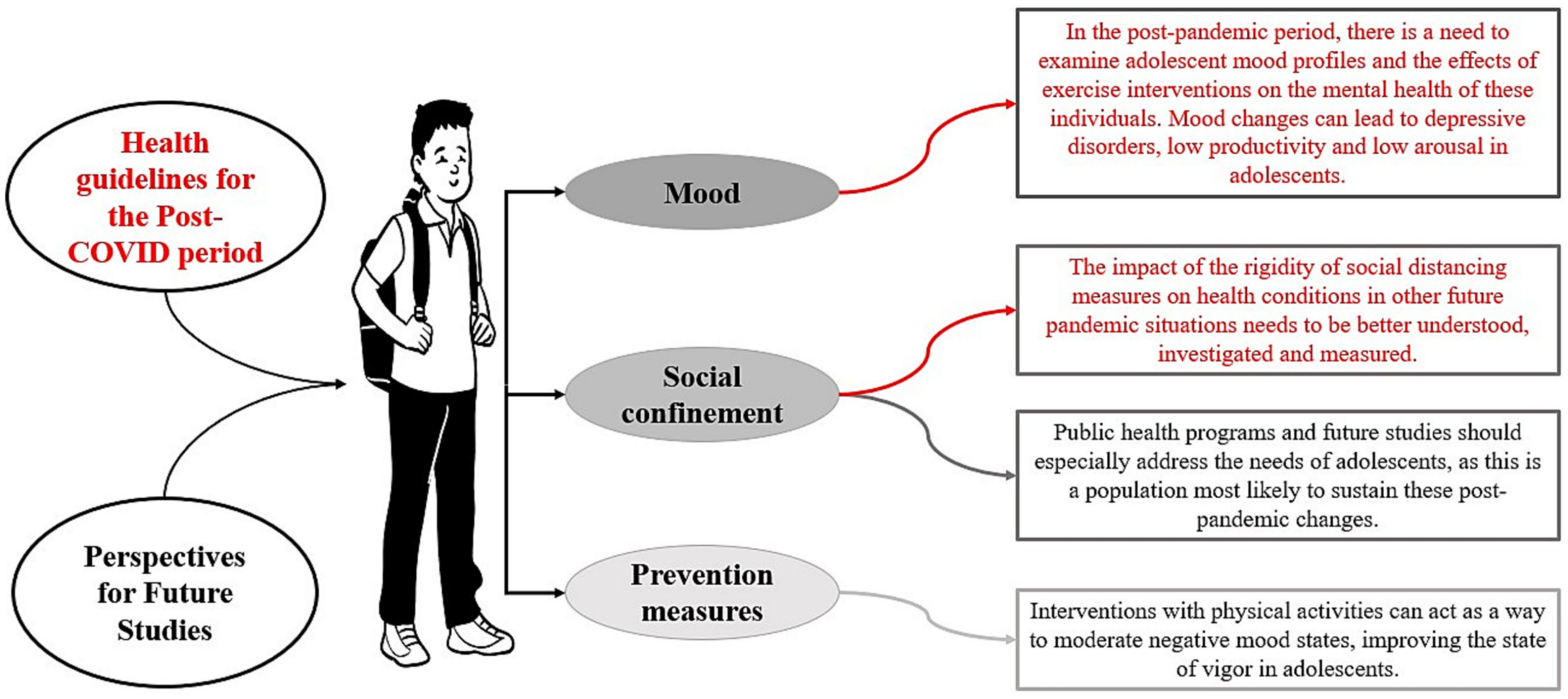
Health Guidelines for Adolescents in the Post-COVID Period.

As prevention measures, it is noteworthy that interventions with physical activities can act as a way to moderate negative mood states, improving the state of vigor in adolescents (Figure 5).

## Discussion

The current study aimed to evaluate the impact of physical activity on the mood states of active and insufficiently active Italian adolescents in the city of Cassino, Italy, during the pandemic and the relationships with sleep quality, sex, and academic performance, analyzing guidelines for the post-COVID-19 scenario. The results show that physically active adolescents have a better mood profile when compared to those who do not perform physical activity, with a statistically significant difference in vigor.

Pandemics are known to cause stress, worry, helplessness, and risky social and behavioral problems among children and adolescents (Meherali et al., 2021). The increased use of smartphones in these situations, associated with an exacerbated sedentary lifestyle, can lead to increased anger, mental confusion, depressive mood, fatigue, tension, and depression symptoms, as well as lower levels of vigor (Pereira et al., 2020). Among adolescents, low mood levels can lead to depression, tension, lack of enthusiasm, efficiency, and productivity, reducing their academic performance and negatively affecting their daily life (Samełko et al., 2018; Huang et al., 2020). The impact of COVID-19 on the mental health of children and adolescents is very worrying, as this is a population with a high probability of persistence of these changes in the post-pandemic period (Meherali et al., 2021).

The literature points out that physical activity has been correlated with psychological health and can improve the psychological state of children and adolescents under the influence of COVID-19 (Okuyama et al., 2021). In the same vein, the linear regression analysis performed in our study showed that physical activity is a factor that explains the increase in vigor in Italian adolescents and that for each minute of physical activity practiced, the score in this mood state increases by 0.02, that is, the practice of physical activity constituted a protective factor for the mood state of the adolescent. In the current study, we found a weekly average of physical activity, both in physical education class and outside of it, reported in minutes, of 276.6 (± 217.6) minutes. However, 32% of adolescents did not comply with the WHO recommendations, with a weekly average of only 79 min of physical activity, a worrying fact from the point of view of mental health. The observed differences in physical activity levels were closely linked to more negative mood profiles, suggesting that insufficient physical activity may be a significant factor in exacerbating mood disturbances among adolescents. These results suggest a potential bidirectional relationship between sleep quality and physical activity, where each factor positively reinforces the other, leading to improved mood states in adolescents. Although our results indicate a strong link between physical activity and mood states, it is also possible that other factors, such as dietary habits or social support, may play a mediating role. Our results are consistent with Li et al. (2022), who also found a positive relationship between physical activity and mood in adolescents, and our study further highlights the critical role of sleep quality as a moderator.

Other studies have investigated the effects of physical activity on mood in students. Roh et al. (2018) sought to investigate the effects of regular taekwondo training on students and observed that physical activity was positively associated with improved vigor, as well as lower levels of tension and depression after the intervention. The study by Znazen et al. (2022), included an intervention with strength exercises for students and observed that the moderate-intensity group was positively associated with an improvement in vigor. On the other hand, the high-intensity group presented higher levels of fatigue and tension compared to the other groups. Thus, exercise intensity deserves greater attention in future research on mood and physical activity in adolescents.

There is still no clear consensus on the relative importance of psychological and physiological hypotheses in explaining the relationship between physical activity and improved mood. The most appropriate model will probably be psychobiological, combining both perspectives (Peluso and Guerra de Andrade, 2005). However, for this model to be clearly defined, a deeper understanding of the mechanisms that link physical activity to these hypotheses is necessary, as well as of the processes that associate these hypotheses with improved mood. With this knowledge, it is hoped that a model will be developed in which psychological and biological factors interact in a precise and interconnected manner, varying according to environmental stimuli and the psychological and biological characteristics of each person (Paluska and Schwenk, 2000; Peluso and Guerra de Andrade, 2005).

On the other hand, it is already well established that the beneficial effects of physical activity on the body are largely linked to biological mechanisms, such as increased cerebral blood flow and maximum oxygen consumption capacity, improved oxygen supply to brain tissue, decreased muscle tension and increased levels of endocannabinoid receptors in the blood (Mandolesi et al., 2018). In addition, neuroplasticity processes, including changes in neurotransmitters, play a fundamental role in different mental states (Mandolesi et al., 2018). For example, physical activity increases concentrations of serotonin and beta-endorphins, such as anandamide (Mandolesi et al., 2018).

Within the scope of psychological hypotheses, it is proposed that physical activity improves well-being through factors such as a sense of control, competence, self-efficacy, improved self-concept and self-esteem, and positive social interactions, in addition to providing fun and pleasure (Weinberg and Gould, 2024).

This comprehensive summary of the literature can help us explain the results found in the present study regarding the impacts of physical activity on the mood states of active Italian adolescents, compared to insufficiently active ones. Since, when physical activity practices are carried out, the current chains of changes occur, both psychological and biological in the practitioners.

In the analyses carried out in the current study, we found that when the mood state was analyzed by sex, girls had a worse mood state in relation to boys, with greater tension, depression, anger, fatigue, and mental confusion, and worse vigor. This finding is similar to that of Terry et al. (2021), where the Brunel Mood Scale (BRUMS) was completed by 15,692 participants through the in the Mood website. The results showed that women reported negative mood profiles more often than men, suggesting that refinement of existing tables of normative data for BRUMS should be considered. Male adolescents were represented in the iceberg mood profiles and surface profiles, while female adolescents were represented in the inverse Everest, reverse iceberg, shark fin, and submerged profiles (Terry et al., 2021). The worse mood outcomes of adolescent girls may be justified by female hormonal alterations during the complex developmental stage of puberty. These results suggest that although puberty is a critical period for mood development, hormonal changes, particularly in estradiol, may explain variability in mood outcomes during puberty, but are only one piece of a larger puzzle, with genetic and psychosocial factors also contributing to mood disorders in adolescent girls (Viner, 2015; Hazell et al., 2023). These data can guide clinicians and parents to consider alternative explanations for mood disorders in adolescents (Hazell et al., 2023).

Another factor that has been shown to affect adolescent girls’ moods today that was not controlled for in this study may be related to social media. Recent research shows that social media significantly impacts adolescent girls’ body image and mood, often leading to negative psychological outcomes (American Psychological Association, 2023). Many girls compare their appearance to idealized and heavily edited images, particularly on highly visual platforms such as Instagram and TikTok. These comparisons can increase body dissatisfaction, which has been linked to increased depressive symptoms and disordered eating behaviors (Glaser et al., 2024). Girls who spend more time on these platforms are also more likely to engage in passive social media use, which exacerbates these effects. These outcomes often lead to a cycle of negative body image and poor mental health (Papageorgiou et al., 2022). While some movements like body positivity are attempting to combat these trends, the pervasive nature of appearance-based comparisons on social media remains a key factor in low mood outcomes among adolescent girls (American Psychological Association, 2023).

When comparing the mood between good and poor sleep quality in adolescents, our study showed that adolescents with a worse mood also have poor sleep quality. This result is in line with the research by Asarnow and Mirchandaney (2021), where a review of the literature on mood disorders and sleep disorders in children and adolescents was carried out. Research suggests that sleep plays an important role in the development, progression, and maintenance of mood disorder symptoms among children and adolescents. In the study by Hysing et al. (2022), the prevalence of sleep disorders diagnosed among adolescents seeking mental health services was 0.6%, resulting in an estimated prevalence of 0.07% of the population. The questionnaire-based assessment of insomnia indicated that it was highly prevalent across all disorders, compared to a reference group of adolescents who were not under mental health care (Hysing et al., 2022). Sleep problems appear to be prevalent among adolescents, especially among those diagnosed with mental health disorders or mood disorders. These data deserve attention from parents and health professionals for better management of these alterations.

In the comparisons between the mood states and the perception of academic performance of the adolescents, we found that adolescents who perceive themselves as having insufficient academic performance have a significantly worse mood state, in tension, depression, anger, and mental confusion, when compared to adolescents who perceive themselves as excellent in terms of their academic performance. In this case, we verified the submerged profile of negative mood of these adolescents who perceive their academic performance as insufficient. Similar results were found in the study by López-López et al. (2021), where 13,599 British adolescents aged 11 and 18 were investigated. The authors observed that higher depressive symptoms were associated with lower academic performance. Equitably, a systematic review found evidence to suggest positive associations between PA and academic achievement. However, data on the type, quantity, frequency, and duration of PA and their relationships with mood state still need to be explored (López-López et al., 2021).

Knowing the characteristics of students’ mood states in a period of the COVID-19 pandemic and how these can be influenced by exercise is of paramount importance for health and education professionals, as well as for the parents of these young people. The results of the present study contribute to the development of early actions to curb the impact of a sedentary lifestyle on mental health, thinking about a post-COVID-19 scenario.

### Limitations and future studies

The current study presents some limitations, such as the cross-sectional design of the study, which does not allow causal inferences from the data found. In addition, the data were self-reported, which may be affected by memory bias and interpretation of the participants. While the cross-sectional design of this study limits the ability to infer causality, the robust sample size and the use of validated measurement instruments provide confidence in the observed associations. Another limitation of this study is the geographic concentration of the sample, the lack of sociodemographic data, and the fact that height and weight were self-reported, which may limit the generalization of the findings to broader populations. Future research should consider a more diverse sample to validate these results. Considering the population studied, other variables would be important to include in the control in future studies, as they may affect mood states, such as diet, family history, and screen time.

Despite the low number of adult students that made up the sample and the fact that they are unlikely to directly impact the results of the present study, for future studies, separating the samples according to different age groups would allow the control of confounding variables since young individuals show age-dependent responses to short- and long-term physical activities when compared to adults, which could show us different results with a considerable number for comparison between samples (Boisseau and Delamarche, 2000). Another point that limits the present study is the control over the level of student involvement in student sports, since within the present sample, we could have athletes of some modality. This is also a point to be taken into consideration in future studies since athletes have a much higher time in minutes of exercise than the general population. In addition, more studies are needed to clarify which types, intensity, and dosage of physical activity bring the best results, since the present study was unable to measure these. Finally, future studies should investigate the long-term effects of combined physical activity and sleep interventions on mood states, as well as explore potential differences between different cultural contexts.

### Strengths, innovations, and applications

The results of the current study contribute to the timely development of measures that reduce the impact of a sedentary lifestyle on mental health, considering the post-COVID-19 scenario. The PA recommendation may act as a way to moderate potential levels of negative mood states, as this population is highly likely to sustain these post-pandemic changes. Therefore, with the present findings, the recommendations regarding PA practices for adolescents can be reinforced in the main guidelines for the post-COVID-19 scenario. These findings have important policy implications, suggesting that schools and community programs should prioritize physical activity and sleep education as integral components of adolescent mental health initiatives. This study not only corroborates previous findings on the link between physical activity and mood states but also introduces novel insights into the moderating role of sleep quality, particularly in adolescent populations post-COVID-19.

## Conclusion

The current study significantly enhances our understanding of how physical activity influences adolescent mood states in a post-COVID-19 context. The findings reveal that physical activity seems to be associated with positive mood, and participants who performed physical activities according to WHO guidelines showed better vigor. The students who reported physical activity beyond the recommended level showed even better moods. We found that the mood of girls is worse when compared to the mood of boys and that adolescents who perceive themselves as having insufficient academic performance present a significantly worse mood state, in tension, depression, anger, and mental confusion. The results showed that adolescents with a worse mood have poor sleep quality.

Thus, the present findings strengthen the recommendations on PA practices for adolescents in the main guidelines for the post-COVID-19 scenario; for each minute of physical activity practiced, the score in the state of mood vigor increases by 0.02. The PA recommendations can act as a way to mitigate possible levels of negative mood states, since this population has a high probability of persistence of these changes in the post-pandemic period, and there is a tendency for adolescents at the global level to increasingly stop performing PA practices, according to data from the World Health Organization.

Given the robust association between physical activity and mood improvement, it is crucial that educators, parents, and policymakers prioritize physical activity as a key component of adolescent well-being programs. However, further studies are needed to clarify which types, intensity, and dosage of physical activity bring the most relevant results for mood states.

## Data Availability

The raw data supporting the conclusions of this article will be made available by the authors, without undue reservation.
